# Hyaluronic acid is associated with organ dysfunction in acute respiratory distress syndrome

**DOI:** 10.1186/s13054-017-1895-7

**Published:** 2017-12-14

**Authors:** Anthony J. Esposito, Pavan K. Bhatraju, Renee D. Stapleton, Mark M. Wurfel, Carmen Mikacenic

**Affiliations:** 1 Department of Medicine, Division of Pulmonary and Critical Care Medicine, University of Washington, 325 Ninth Avenue, Box 359640, Seattle, WA 98104 USA; 2 Department of Medicine, Division of Pulmonary and Critical Care Medicine, University of Vermont College of Medicine, Burlington, VT USA

**Keywords:** Acute respiratory distress syndrome, Glycosaminoglycan, Hyaluronic acid, Lung injury score, Organ dysfunction, Sequential organ failure assessment score

## Abstract

**Background:**

Hyaluronic acid (HA), an extracellular matrix component, is degraded in response to local tissue injury or stress. In various animal models of lung injury, HA has been shown to play a mechanistic role in modulating inflammation and injury. While HA is present in the lungs of patients with acute respiratory distress syndrome (ARDS), its relationship to patient outcomes is unknown.

**Methods:**

We studied 86 patients with ARDS previously enrolled in the Phase II Randomized Trial of Fish Oil in Patients with Acute Lung Injury (NCT00351533) at five North American medical centers. We examined paired serum and bronchoalveolar lavage fluid (BALF) samples obtained within 48 hours of diagnosis of ARDS. We evaluated the association of HA levels in serum and BALF with local (lung injury score (LIS)) and systemic (sequential organ failure assessment score (SOFA)) measures of organ dysfunction with regression analysis adjusting for age, sex, race, treatment group, and risk factor for ARDS.

**Results:**

We found that both day-0 circulating and alveolar levels of HA were associated with worsening LIS (*p* = 0.04 and *p* = 0.003, respectively), particularly via associations with degree of hypoxemia (*p* = 0.02 and *p* < 0.001, respectively) and set positive end-expiratory pressure (*p* = 0.01 and *p* = 0.02, respectively). Circulating HA was associated with SOFA score (*p* < 0.001), driven by associations with the respiratory (*p* = 0.02), coagulation (*p* < 0.001), liver (*p* = 0.006), and renal (*p* = 0.01) components. Notably, the alveolar HA levels were associated with the respiratory component of the SOFA score (*p* = 0.003) but not the composite SOFA score (*p* = 0.27).

**Conclusions:**

Elevated alveolar levels of HA are associated with LIS while circulating levels are associated with both lung injury and SOFA scores. These findings suggest that HA has a potential role in both local and systemic organ dysfunction in patients with ARDS.

**Electronic supplementary material:**

The online version of this article (doi:10.1186/s13054-017-1895-7) contains supplementary material, which is available to authorized users.

## Background

Acute respiratory distress syndrome (ARDS) is a leading cause of acute respiratory failure in critically ill medical and surgical patients. Despite significant advances in our understanding of the pathophysiology, management, and outcomes of ARDS, the morbidity and mortality remains unacceptably high, with a recent pooled estimate of mortality at 43%, ranging from 26 to 58% [1–4]. The pathogenesis of ARDS involves a complex network of cytokines and other pro-inflammatory compounds and the innate and adaptive immune system, leading to alveolar epithelial and endothelial damage [5]. Knowledge of the mechanisms underlying the pathogenesis of ARDS may identify novel therapeutic targets.

Increased extracellular matrix (ECM) turnover is a prominent feature of tissue injury in ARDS. Previous studies have shown that failure to remove ECM degradation products from the site of injury results in unremitting inflammation [6]. Hyaluronic acid (HA)—a nonsulfated glycosaminoglycan composed of repeating disaccharide units of D-glucuronic acid and N-acetyl glucosamine—is an important component of the ECM and is ubiquitously distributed in the lung parenchyma of humans [7]. Although HA has traditionally been regarded as a structural molecule, it has been shown to undergo dynamic regulation and possess bioactive properties [8].

The concentration of HA in the interstitium is a function of the equilibrium between synthesis and degradation, directly influencing circulating and tissue HA levels. The balance between these two processes is altered in many disease states. Increased HA deposition has been observed in the lung tissue of rats with bleomycin-induced lung injury [9] and in humans with ARDS [10, 11], whereas increased HA synthesis has been described in non-human primates with ARDS [12]. Hypoxemia alone has been shown to increase HA synthesis and hyaluronidase activity [13] and to upregulate the expression of the endogenous HA receptor, CD44 [14]. In bleomycin-induced alveolar injury in rats, an association between accumulation of HA in the lung interstitium and pulmonary edema has been demonstrated [9]. Furthermore, serum HA has been proposed as a prognostic factor in patients with ARDS undergoing extracorporeal CO_2_ removal [15] and HA levels have been associated with other forms of organ injury particularly in sepsis [16, 17]. Urinary levels of HA have been shown to predict the eventual development of acute kidney injury and in-hospital mortality [18, 19]. Although these studies suggest a role for HA in the pathogenesis of lung injury and systemic inflammation, the mechanisms responsible for the observed associations between HA and patient outcomes are not well-understood.

Given the role of HA in pathologic inflammation in animal models of lung injury and clinical correlations in humans, we sought to determine the relationship between HA levels in the vascular and alveolar compartments and clinical outcomes. We hypothesized that increasing levels of HA would be associated with ARDS-related outcomes. We performed a secondary analysis of a prior randomized controlled trial in ARDS to test this hypothesis.

## Methods

### Study population

Patients meeting criteria for acute lung injury as defined by the American-European Consensus Conference in 1994 [20] were enrolled between 2006 and 2008 at five North American centers for a phase II randomized placebo-controlled trial of omega-3 fatty acids [21] (ClinicalTrials.gov Identifier, NCT00351533). Of note, the enrolled subjects also met criteria for the more recent “Berlin” definition of ARDS [22]. Institutional review boards at each participating institution (University of Washington Medical Center in Seattle, Washington; St. Michael’s Hospital in Toronto, Ontario; St. Alphonsus Medical Center in Boise, Idaho; and Fletcher Allen Health Care in Burlington Vermont) approved the study protocol, and written informed consent was obtained from legal next of kin. Subjects were randomized in a 1:1 ratio to receive enteral fish oil (9.75 g of eicosapentaenoic acid and 6.75 g of docosahexanoic acid) or 0.9% saline within 48 hours of ARDS onset. Subjects received the study drug for 14 days or until ICU discharge, death or 48 hours of unassisted breathing. Bronchoalveolar lavage fluid (BALF) was collected in a standardized manner in which a fiber optic bronchoscope was wedged in the right middle lobe or lingua and five separate 30-mL aliquots of normal saline were instilled and recovered by suction. Bronchoscopy was performed on enrollment (day 0), days 4 ± 1 and 8 ± 1 in all patients who were alive and still intubated (day 4 BALF n = 64 and day 8 BALF n = 39) [21, 23]. Clinical data including Acute Physiology and Chronic Health Evaluation (APACHE) II score [24], serum creatinine, and lung injury score (LIS; Additional file 1) [25], and outcomes such as sequential organ failure assessment (SOFA) score (Additional file 2) [26], ventilator-free days (VFDs), and 28-day mortality were ascertained. There was no effect of the fish oil intervention on the primary outcome of change in BALF IL-8 levels nor was there any significant effect on secondary outcomes including mortality, VFDs, ICU-free days, or worst multiple organ dysfunction score through day 28.

The data reported in the current study includes results from 86 of the original 89 patients enrolled, as 3 patients were excluded due to immunosuppression. Sepsis was defined by documentation of sepsis by ICU providers or if the patient met two of four systemic inflammatory response syndrome criteria and had suspected infection at study enrollment [27]. ARDS was further classified as direct (i.e., pneumonia, aspiration, inhalation injury, near-drowning, lung contusion) vs indirect (i.e., extrapulmonary sepsis, nonthoracic trauma) based on the underlying clinical risk for ARDS [28–30].

### Measurement of HA

We determined both serum (circulating) and BALF (alveolar) levels of HA by an enzyme-linked immunosorbent assay (ELISA; R&D Systems, Minneapolis, MN, USA) according to the manufacturer’s protocol.

### Statistical analysis

We compared BALF and serum HA concentrations between placebo and fish-oil-treated groups using the Mann-Whitney test. We tested correlation between log_10_-transformed serum and BALF levels of HA at each sampling interval using Pearson’s correlation test. Our primary analysis tested for associations between HA levels and LIS as a marker of lung injury severity. We also tested for associations between HA and severity of ARDS based on VFDs and the Berlin definition [30]. We then tested associations between HA levels and measures of systemic organ dysfunction (SOFA score (maximum over initial 7 days), 28-day mortality). We tested for associations between log_10_-transformed day-0 HA levels and outcome measures using multiple linear regression (LIS, VFDs, Berlin definition, SOFA) or multiple logistic regression (mortality). We adjusted for age, sex, race, treatment group (omega-3 fatty acid vs placebo), and ARDS risk factor (direct vs indirect), which were covariates chosen based on previous publications [20, 28–30]. Finally, in exploratory analyses, we tested associations between HA levels and the various components of the LIS and SOFA score. VFD measurements were organized into quartiles, given the skewed distribution. We used the maximum SOFA score during the first 7 days of the trial. We performed analyses using STATA XIV (College Station, TX, USA).

## Results

Characteristics of enrolled patients are shown in Table 1. Eighty-six patients affected by ARDS were studied. The population consisted of predominately white (88%) men (63%) with a mean age of 50 years. In 47 (55%) of the patients, direct lung injury was the risk factor for ARDS (direct ARDS), whereas the other 39 (45%) had an extrapulmonary (indirect ARDS) risk factor for the syndrome (i.e., extrapulmonary sepsis or nonthoracic trauma). A majority of patients (57%) had moderate ARDS while 29% and 12% had mild and severe ARDS, respectively. The average highest SOFA score within 7 days was 9 ± 4: 56 (65%) of the patients met criteria for sepsis at study enrollment. The majority of the patients (85%) were still alive after 28 days. Comparing subjects treated with placebo vs fish oil, there was no difference in day-0 BALF HA (median (IQR) 63 (23–216) ng/ml vs 56 (17–215) ng/mL; *p* = 0.96) or day-0 serum HA concentration (median (IQR) 119 (53–245) ng/mL vs 148 (67–242) ng/mL; *p* = 0.57]. Day-0 (study enrollment) serum HA levels (median 125.85 ng/mL, interquartile range (IQR) 55.81–241.58 ng/mL) and BALF HA levels (median 62.00 ng/mL, IQR 20.78–215.95 ng/mL) were positively correlated (*r* = 0.26, *p* = 0.02; Additional file 3). However, this correlation was not observed in patients who survived and remained intubated on day 4 (*p* = 0.47) or day 8 (*p* = 0.64).

**Table 1:** Demographics and clinical characteristics of patients at study enrollment

Characteristic	Values in patients (n = 86)
Age (years), mean ± SD	50 ± 17
Male sex, *n* (%)	54 (63)
Race, *n* (%)	
White	76 (88)
African American	3 (4)
Other^a^	7 (8)
Co-morbidities, *n* (%)	
Fish oil treatment, *n* (%)	38 (44)
Diabetes mellitus	18 (21)
Liver cirrhosis	6 (7)
Chronic kidney disease	2 (2)
ARDS risk factor, *n* (%)	
Direct^*b*^	47 (55)
Indirect^*c*^	39 (45)
ARDS classification of severity^d*,*e^, *n* (%)	
Mild	25 (29)
Moderate	49 (57)
Severe	10 (12)
APACHE II score, mean ± SD	22 ± 6
Sepsis, *n* (%)	56 (65)
Day-0 serum HA (ng/mL), median (IQR)	125.85 (55.81–241.58)
Day-0 BALF HA (ng/mL), median (IQR)^f^	62.00 (20.78–215.95)
Lung injury score, mean ± SD	3 ± 1
Sequential organ failure assessment score, mean ± SD^g^	9 ± 4
28-Day mortality, *n* (%)	13 (15)

*ARDS* acute respiratory distress syndrome, *APACHE* Acute Physiology and Chronic Health Evaluation, *HA* hyaluronic acid, *BALF* bronchoalveolar lavage fluid

^a^Includes American Indian, Asian, Pacific Islander, and unknown

^b^Direct ARDS was defined as pneumonia, aspiration, inhalation injury, near drowning, or lung contusion as the risk factor for ARDS

^c^Indirect ARDS was defined as extrapulmonary sepsis or nonthoracic trauma as the risk factor for ARDS

^d^Severity of ARDS was classified by the “Berlin” definition [22]

^e^Two patients did not have day-1 ratio of arterial oxygen partial pressure to fractional inspired oxyge (P/F) ratios recorded

^f^Two patients did not have day-0 BALF samples (n = 84)

^g^Highest sequential organ failure assessment score in first 7 days of study

### HA levels are associated with measures of local organ injury

**Fig. 1:**
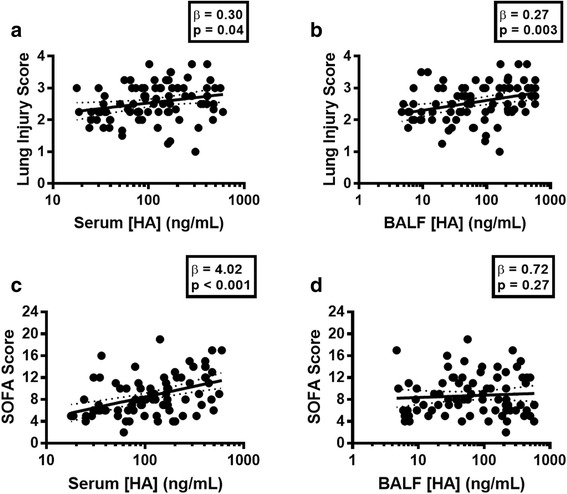
Compartmentalized hyaluronic acid levels are differentially associated with measures of local and systemic organ injury. Elevated alveolar levels of hyaluronic acid are associated with lung injury score (LIS) (**a**) but not sequential organ failure assessment (SOFA) score (**b**), while circulating levels are associated with both (**c**, **d**). [HA], concentration of hyaluronic acid. β values represent a change in units of either LIS (**a**, **b**) or SOFA (**c**, **d**) score per tenfold increase in [HA]. Solid lines represent regression lines determined via linear regression analyses, while hashed lines represent the 95% confidence interval of the regression line

We tested for associations between HA levels in serum and BALF and measures of local lung injury severity. Considering the concentration of HA at study enrollment (day 0) and adjusting for age, sex, race, treatment group, and ARDS risk factor, a tenfold increase in day-0 serum HA concentration was associated with an increase of 0.30 in LIS (*p* = 0.04; Fig. 1a and Table 2). A tenfold increase in BALF HA concentration was associated with a 0.27 increase in LIS (*p* = 0.003; Fig. 1b and Table 2). Both serum and BALF HA levels were associated with severity of ARDS as defined by the Berlin criteria (*p* = 0.001; Additional file 4). Neither serum (*p* = 0.14) nor BALF (*p* = 0.47) HA levels were associated with quartile of VFDs (Table 2).

**Table 2:** Association of early (day 0) hyaluronic acid levels with degree of end organ dysfunction

Outcome	Source	β (95% CI)^a^	*p*	β (95% CI)^b^	*p*
Lung injury score	Serum	0.35 (0.05–0.65)	0.02	0.30 (0.02–0.57)	0.04
	BALF^c^	0.31 (0.11–0.50)	0.002	0.27 (0.09–0.45)	0.003
Ventilator-free days^d^	Serum	-0.59 (-1.21–0.03)	0.06	-0.47 (-1.10–0.15)	0.14
	BALF^c^	-0.09 (-0.51–0.33)	0.67	-0.15 (-0.58–0.27)	0.47
SOFA^e^	Serum	3.89 (2.18–5.61)	<0.001	4.02 (2.30–5.74)	<0.001
	BALF^c^	0.42 (-0.85–1.69)	0.52	0.72 (-0.56–2.00)	0.27

*SOFA* sequential organ failure assessment score, *BALF* bronchoalveolar fluid,

^a^Linear regression between log_10_-transformed day-0 hyaluronic acid concentration and respective outcome measure (in units of lung injury score, quartiles of ventilator-free days, or units of SOFA)

^b^Multiple linear regression adjusted for age, sex, race, treatment group, and acute respiratory distress syndrome etiology

^c^Two patients did not have day-0 BALF samples (n = 84)

^d^Ventilator-free days were analyzed as quartiles, given the skewed distribution

In exploratory analyses, we sought to identify which component(s) of the LIS was responsible for the associations observed with the composite score. We found that both serum and BALF HA levels were strongly associated with the degree of hypoxemia (*p* = 0.02 and *p* < 0.001, respectively) and with the set positive end-expiratory pressure (PEEP) (*p* = 0.01 and *p* = 0.02, respectively; Additional file 5 and Table 3). Neither serum nor BALF HA levels were associated with the number of affected quadrants on a chest radiograph (*p* = 0.21 and *p* = 0.53, respectively) or respiratory system compliance (*p* = 0.89 and *p* = 0.69, respectively; Table 3 and Additional file 5). These data demonstrate that both circulating and alveolar HA levels are associated with severity of lung injury, primarily through associations with the degree of hypoxemia and the set PEEP.

**Table 3:** Association of early (day 0) hyaluronic acid levels with components of LIS

Lung injury score component^a^	Source	β (95% CI)^b^	*p*	β (95% CI)^c^	*p*
Chest radiograph^d^	Serum	-0.15 (-0.54–0.24)	0.44	-0.25 (-0.63–0.14)	0.21
	BALF^e^	-0.05 (-0.30–0.20)	0.69	-0.08 (-0.33–0.17)	0.53
Hypoxemia^f^	Serum	0.66 (0.19–1.12)	0.006	0.57 (0.11–1.03)	0.02
	BALF^e^	0.57 (0.28–0.86)	<0.001	0.56 (0.28–0.85)	<0.001
Positive end-expiratory pressure	Serum	0.77 (0.16–1.38)	0.01	0.76 (0.16–1.35)	0.01
	BALF^e^	0.57 (0.17–0.96)	0.006	0.46 (0.07–0.85)	0.02
Respiratory system compliance^g^	Serum	0.005 (-0.35–0.36)	0.98	0.02 (-0.31–0.36)	0.89
	BALF^e^	0.09 (-0.14–0.32)	0.43	0.04 (-0.17–0.26)	0.69

*LIS* lung injury score, *BALF* bronchoalveolar fluid

^a^See Additional file 1: Table S1 for scoring of composite LIS via individual end-organ components

^b^Linear regression between log_10_-transformed day-0 hyaluronic acid concentration and respective outcome measure (in units of LIS)

^c^Multiple linear regression adjusted for age, sex, race, treatment group, and acute respiratory distress syndrome etiology

^d^One patient did not have a chest radiograph available to interpret

^e^Two patients did not have day-0 BALF samples (n = 84)

^f^Two patients did not have blood gas data available (n = 84)

^g^Nine patients did not have compliance data available (n = 77)

### HA levels are associated with measures of organ dysfunction

We next tested for associations between HA and systemic measures of organ dysfunction. Considering the concentration of HA at study enrollment (day 0) and adjusting for age, sex, race, treatment group, and ARDS risk factor, we found that a tenfold increase in serum HA concentration was associated with an increase of 4 points in SOFA score (*p* < 0.001; Fig. 1c and Table 2). BALF HA concentration was not associated with increasing SOFA score (*p* = 0.27; Fig. 1d and Table 2). Neither serum HA (*p* = 0.21) nor BALF HA (*p* = 0.32) concentration were associated with 28-day mortality (Additional file 6).

In exploratory analyses, we sought to identify which component(s) of the SOFA score was responsible for the strong association observed between serum HA levels and the composite score. We found that serum HA levels were associated with the respiratory (*p* = 0.02), coagulation (*p* < 0.001), liver (*p* = 0.006), and renal (*p* = 0.01) components of the SOFA score but not the cardiovascular (*p* = 0.09) or neurologic (*p* = 0.18) components (Table 4 and Additional file 7). BALF HA levels were solely associated with the respiratory (*p* = 0.003) component of the SOFA score (Table 4 and Additional file 7). These data demonstrate that circulating HA levels are strongly associated with multi-organ dysfunction, while alveolar HA levels are associated only with the respiratory component of the SOFA score.

**Table 4:** Association of early (day 0) hyaluronic acid levels with components of the SOFA score

SOFA component^a^	Source	β (95% CI)^b^	*p*	β (95% CI)^c^	*p*
Respiratory	Serum	0.45 (0.10–0.80)	0.01	0.42 (0.06–0.78)	0.02
	BALF^d^	0.37 (0.14–0.60)	0.002	0.36 (0.13–0.60)	0.003
Coagulation	Serum	0.93 (0.48–1.37)	<0.001	1.04 (0.60–1.47)	<0.001
	BALF^d^	-0.12 (-0.44–0.20)	0.45	-0.08 (-0.40–0.25)	0.65
Liver^e^	Serum	0.77 (0.21–1.34)	0.007	0.78 (0.22–1.33)	0.006
	BALF^d^	-0.02 (-0.44–0.20)	0.93	0.08 (-0.30–0.54)	0.69
Cardiovascular	Serum	0.79 (-0.01–1.60)	>0.05	0.70 (-0.12–1.5)	0.09
	BALF^d^	0.22 (-0.32–0.76)	0.42	0.30 (-0.24–0.85)	0.27
Neurologic	Serum	0.31 (-0.34–0.96)	0.34	0.44 (-0.21–1.1)	0.18
	BALF^d^	0.01 (-0.42–0.44)	0.95	0.09 (-0.35–0.52)	0.69
Renal	Serum	0.60 (0.16–1.05)	0.01	0.57 (0.12–1.02)	0.014
	BALF^d^	-0.08 (-0.39–0.23)	0.60	-0.05 (-0.36–0.26)	0.74

*SOFA* sequential organ failure assessment, *BALF* bronchoalveolar lavage fluid

^a^See Additional file 2: Table S2 for scoring of composite SOFA score and individual components

^b^Linear regression between log_10_ transformed day 0 hyaluronic acid concentration and respective outcome measure (in units of SOFA score)

^c^Multiple linear regression adjusted for age, sex, race, treatment group, and acute respiratory distress syndrome etiology

^d^Two patients did not have day-0 BALF samples (*n* = 84)

^e^Eight patients did not have total bilirubin data available (*n* = 78)

*SOFA* Sequential organ failure assessment score

## Discussion

Previous evidence supports a role for HA in lung injury and remodeling [9–15]. In this study, we built upon prior studies to show that increasing circulating and alveolar HA levels are associated with severity of lung injury, using simultaneously obtained serum and BALF samples from critically ill patients with ARDS. Alveolar HA appears to be primarily associated with respiratory organ dysfunction rather than systemic organ dysfunction, while circulating HA is associated with systemic organ dysfunction. These results suggest compartmentalized effects of HA in ARDS.

The mechanism by which HA contributes to or is a consequence of local lung injury remains to be determined; however, a few possible mechanistic models have been suggested. For example, low molecular weight forms of HA present during tissue injury can initiate inflammatory responses via Toll-like receptors [31]. Indeed, HA forms a network that maintains epithelial cell integrity during homeostasis [7, 8] and breakdown of this network could contribute to extravasation of protein-rich alveolar fluid. Furthermore, hypoxia induces both the production of HA and the activity of hyaluronidase in vitro, leading to its degradation into a low molecular-weight form [13]. We hypothesize that any one of these mechanisms could theoretically contribute to our observed association between alveolar HA concentration and lung injury severity.

In addition to the local effect in the lung, we hypothesize that HA likely has a systemic effect, as evidenced by our observation of an association between circulating HA levels and SOFA score. We found that in addition to respiratory dysfunction, liver, renal, and coagulation dysfunction drove the strong association observed with circulating HA levels and SOFA score. Previously, it has been shown that urinary HA levels predict the development of acute kidney injury [18, 19]. Furthermore, elevated concentrations of HA have been associated with hepatic dysfunction through decreased hepatic clearance [32]. To our knowledge, however, the association between circulating HA and coagulation dysfunction (i.e., thrombocytopenia) has not been reported. More studies are needed to further characterize these newly described relationships.

Although circulating and alveolar HA levels are strongly associated with SOFA score and LIS, respectively, they are not associated with 28-day mortality or VFDs. One potential reason for these findings may be due to the relatively low mortality rate observed in our cohort (15%) compared to previous population studies for all-comers with ARDS (43%), limiting our power to detect an effect and to generalize results to other populations [1–4]. Another is that VFDs may not be the best measure of lung injury severity, as other factors lead to prolonged mechanical ventilation such as neuromuscular weakness and encephalopathy. Additional work is needed to address these issues to better understand our findings.

This study has important limitations. First, our findings linking HA to severity of organ dysfunction were observed in a single population. In order to validate our findings, we would need to obtain serum and BALF from an independent population of patients with ARDS. Second, our findings do not identify the source of HA in the serum or BALF. HA may be released directly from the ECM of the lungs as a result of tissue degradation and/or remodeling or it may enter into the alveolar space because of increased vascular permeability. Our data showing weak positive correlation between circulating and alveolar HA levels at day 0 and the lack of correlation at days 4 and 8 suggest that HA in the two compartments may be somewhat independent. Third, the mortality in the ARDS patients enrolled in the fish oil study (15%) was lower than that observed in recent trials [33, 34]. This discrepancy limits the generalizability of our findings to a broader population of critically ill patients. In light of these limitations, additional studies will be required in order to generalize these results to patients with more severe ARDS, and to perform sampling of the alveolar fluid to see if HA levels increase prior to the development of lung injury in those at risk.

## Conclusions

In conclusion, this study found that alveolar HA levels in patients recently diagnosed with ARDS are associated with measures of severity of lung injury—suggesting a local effect—while simultaneous circulating HA levels in these patients are associated with measures of other end-organ damage (SOFA score)—suggesting a systemic effect. These findings suggest a strong link between HA and local and systemic organ injury in ARDS. With further characterization of these conclusions, the measurement of HA concentrations may provide diagnostic or prognostic information for patients with ARDS.

## Additional files

Additional file 1: Lung injury score. This table provides the reader with information regarding what components contribute to and how to calculate the composite lung injury score. (DOCX 83 kb)

Additional file 2: Sequential organ failure assessment score. Description: This table provides the reader with information regarding what components contribute to and how to calculate the composite lung injury score. (DOCX 102 kb)

Additional file 3: Circulating and alveolar hyaluronic acid (HA) levels are positively correlated within 48 hours of diagnosis (**A**) of acute respiratory distress syndrome (ARDS) but not at days 4 ± 1 (**B**) or 8 ± 1 (**C**). This figure provides the reader with graphical representation and corresponding statistical analysis of the correlation between HA levels at various sample collection times during the study. As discussed in the text, there is weak correlation at day 0 and no correlation at days 4 and 8, which, per our hypothesis, supports independent compartmentalized effects or processing of HA. (DOCX 262 kb)

Additional file 4: BALF HA and serum HA are associated with severity of ARDS by the Berlin criteria. This figure shows the relationship between HA levels and ARDS severity by the Berlin criteria. (DOCX 39963 kb)

Additional file 5: Both circulating and alveolar hyaluronic acid (HA) levels are associated with hypoxemia (**C**, **D**) and the set positive end-expiratory pressure (PEEP) (**E**, **F**), but not the involved quadrants on a chest radiograph (CXR) (**A**, **B**) or respiratory system compliance (**G**, **H**), components of the lung injury score (LIS). This figure provides the reader with graphical representation and corresponding analysis of the reported data in Table 3 of the main text. (DOCX 259 kb)

Additional file 6: Early (Day 0) hyaluronic acid levels are not associated with mortality. This table provides the reader with analysis of the association between circulating and alveolar hyaluronic acid levels and 28-day mortality. (DOCX 16 kb)

Additional file 7: Both circulating (**A**) and alveolar (**B**) HA levels are associated with the respiratory component of the sequential organ failure assessment (SOFA) score, while circulatory, but not alveolar, HA levels are also associated with coagulation (**C**, **D**), and liver (**E**, **F**) components. Circulatory, but not alveolar, HA levels are also associated with renal components (**K**, **L**). Neither is associated with the cardiovascular (**G**, **H**) or neurologic (**I**, **J**) component. This figure provides the reader with graphical representation and corresponding analysis of the reported data in Table 4 of the text. (DOCX 486 kb)

## Abbreviations

[HA]: Concentration of hyaluronic acid
APACHE II score: Acute Physiology and Chronic Health Evaluation II score
ARDS: Acute respiratory distress syndrome
BALF: Bronchoalveolar lavage fluid
ECM: Extracellular matrix
ELISA: Enzyme-linked immunosorbent assay
HA: Hyaluronic acid
ICU: Intensive care unit
IL: Interleukin
LIS: Lung injury score
PEEP: Positive end-expiratory pressure
SOFA: Sequential organ failure assessment score
VFD: Ventilator-free day
