# Supplementary material for: Hyaluronic acid is associated with organ dysfunction in acute respiratory distress syndrome
Source: Crit Care. 2017 Dec 14;21:304. doi: 10.1186/s13054-017-1895-7 (PMC5729515; doi:10.1186/s13054-017-1895-7)
Supplement: Supplementary file 1 — Lung injury score. This table provides the reader with information regarding what components contribute to and how to calculate the composite lung injury score. (DOCX 83 kb) [file 13054_2017_1895_MOESM1_ESM.docx]

**Additional File 1. Lung Injury Score.**

| **Chest Radiograph** | **Score** |
| --- | --- |
| No alveolar consolidation | 0 |
| Alveolar consolidation confined to 1 quadrant | 1 |
| Alveolar consolidation confined to 2 quadrants | 2 |
| Alveolar consolidation confined to 3 quadrants | 3 |
| Alveolar consolidation in all 4 quadrants | 4 |
| **Hypoxemia: PaO2/FiO2 (mmHg)** | **Score** |
| ≥ 300 | 0 |
| 225-299 | 1 |
| 175-224 | 2 |
| 100-174 | 3 |
| < 100 | 4 |
| **PEEP (cmH2O)** | **Score** |
| ≤ 5 | 0 |
| 6-8 | 1 |
| 9-11 | 2 |
| 12-14 | 3 |
| ≥ 15 | 4 |
| **Respiratory System Compliance (mL/cmH2O)** | **Score** |
| ≥ 80 | 0 |
| 60-79 | 1 |
| 40-59 | 2 |
| 20-39 | 3 |
| ≤ 19 | 4 |

The final score is calculated by summing the components’ individual values and dividing by the number of elements used for the analysis. A score of 0 indicates no lung injury, a score ≤ 2.5 indicates mild-to-moderate lung injury, and a score > 2.5 indicates severe lung injury (22).
