# Supplementary material for: Hyaluronic acid is associated with organ dysfunction in acute respiratory distress syndrome
Source: Crit Care. 2017 Dec 14;21:304. doi: 10.1186/s13054-017-1895-7 (PMC5729515; doi:10.1186/s13054-017-1895-7)
Supplement: Supplementary file 2 — Sequential organ failure assessment score. Description: This table provides the reader with information regarding what components contribute to and how to calculate the composite lung injury score. (DOCX 102 kb) [file 13054_2017_1895_MOESM2_ESM.docx]

**Additional File 2. Sequential Organ Failure Assessment Score.**

| **Respiratory: PaO2/FiO2 (mmHg)** | **Score** |
| --- | --- |
| >400 | 0 |
| 301-400 | 1 |
| 201-300 | 2 |
| 101-200 | 3 |
| ≤100 | 4 |
| **Coagulation: Platelets (x10^3^/μL)** | **Score** |
| >150 | 0 |
| 101-150 | 1 |
| 51-100 | 2 |
| 21-50 | 3 |
| ≤20 | 4 |
| **Liver: Total Bilirubin (mg/dL)** | **Score** |
| <1.2 | 0 |
| 1.2-1.9 | 1 |
| 2.0-5.9 | 2 |
| 6.0-11.9 | 3 |
| ≥12 | 4 |
| **Cardiovascular: Hypotension** | **Score** |
| No hypotension | 0 |
| Mean arterial pressure < 70 mmHg | 1 |
| Dopamine ≤ 5 μg/kg OR Dobutamine at any dose | 2 |
| Dopamine > 5 μg/kg, Epinephrine ≤ 0.1 μg/kg, OR Norepinephrine ≤ 0.1 μg/kg | 3 |
| Dopamine > 15 μg/kg, Epinephrine > 0.1 μg/kg, OR Norepinephrine > 0.1 μg/kg | 4 |
| **Neurologic: Glasgow Coma Scale** | **Score** |
| 15 | 0 |
| 13-14 | 1 |
| 10-12 | 2 |
| 6-9 | 3 |
| < 6 | 4 |
| **Renal: Creatinine (mg/dL) or urine output (mL/day)** | **Score** |
| < 1.2 | 0 |
| 1.2-1.9 | 1 |
| 2.0-3.4 | 2 |
| 3.5-4.9 or < 500 | 3 |
| > 5.0 or < 200 | 4 |

The final score is calculated by summing the components’ individual values (23).
