# Supplementary material for: Hyaluronic acid is associated with organ dysfunction in acute respiratory distress syndrome
Source: Crit Care. 2017 Dec 14;21:304. doi: 10.1186/s13054-017-1895-7 (PMC5729515; doi:10.1186/s13054-017-1895-7)
Supplement: Supplementary file 3 — Circulating and alveolar hyaluronic acid (HA) levels are positively correlated within 48 hours of diagnosis (A) of acute respiratory distress syndrome (ARDS) but not at days 4 ± 1 (B) or 8 ± 1 (C). This figure provides the reader with graphical representation and corresponding statistical analysis of the correlation between HA levels at various sample collection times during the study. As discussed in the text, there is weak correlation at day 0 and no correlation at days 4 and 8, which, per our hypothesis, supports independent compartmentalized effects or processing of HA. (DOCX 262 kb) [file 13054_2017_1895_MOESM3_ESM.docx]

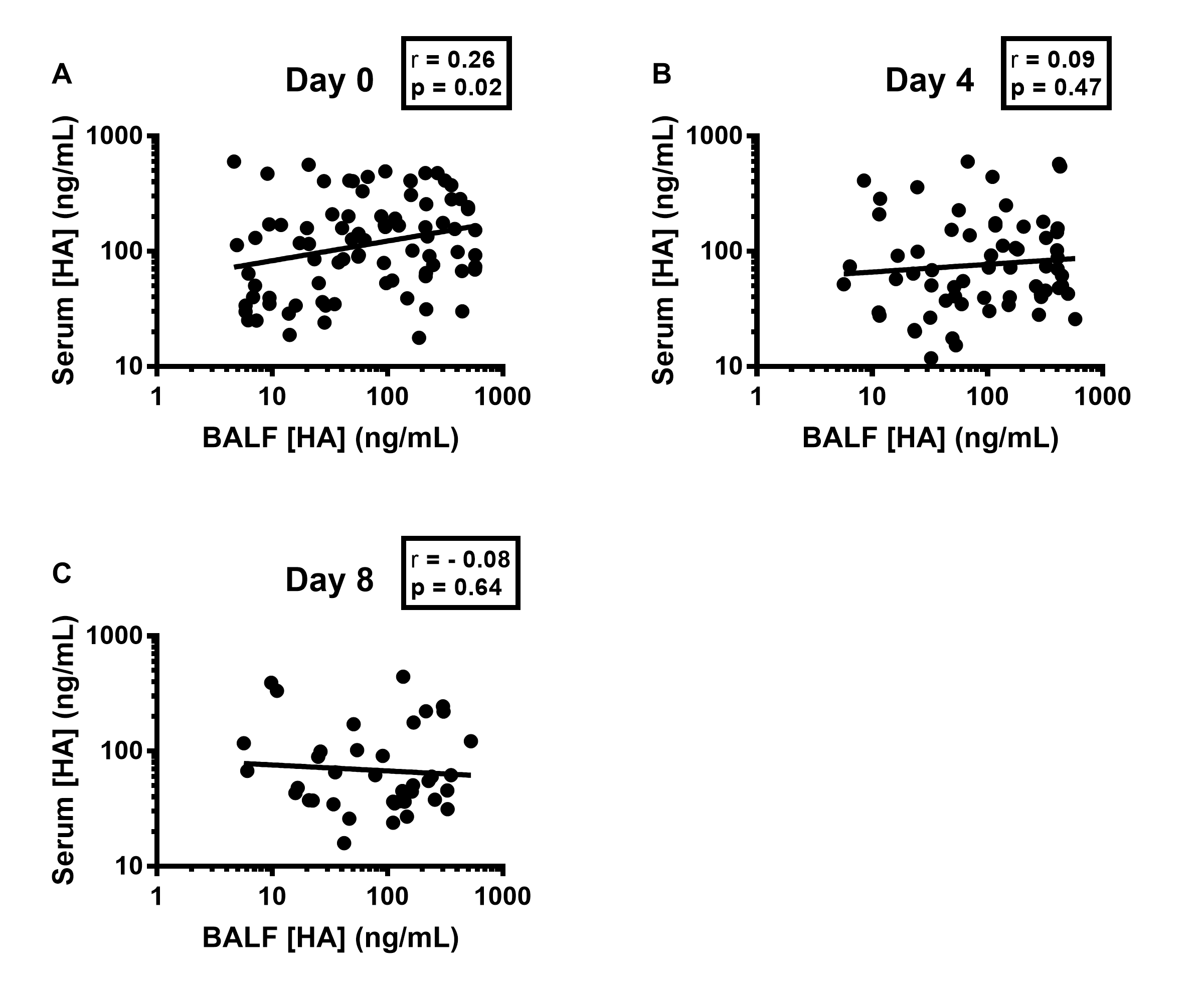


**Additional File 3.** **Circulating and alveolar hyaluronic acid (HA) levels are positively correlated within 48 hours of diagnosis (A) of acute respiratory distress syndrome (ARDS) but not at days 4±1 (B) or 8±1 (C).** [HA] = concentration of hyaluronic acid. Solid lines represent the line of best fit. r values represent the linear dependence between serum and BALF HA concentration as assessed by Pearson’s correlation.
