# Supplementary material for: Hyaluronic acid is associated with organ dysfunction in acute respiratory distress syndrome
Source: Crit Care. 2017 Dec 14;21:304. doi: 10.1186/s13054-017-1895-7 (PMC5729515; doi:10.1186/s13054-017-1895-7)
Supplement: Supplementary file 4 — BALF HA and serum HA are associated with severity of ARDS by the Berlin criteria. This figure shows the relationship between HA levels and ARDS severity by the Berlin criteria. (DOCX 39963 kb) [file 13054_2017_1895_MOESM4_ESM.docx]

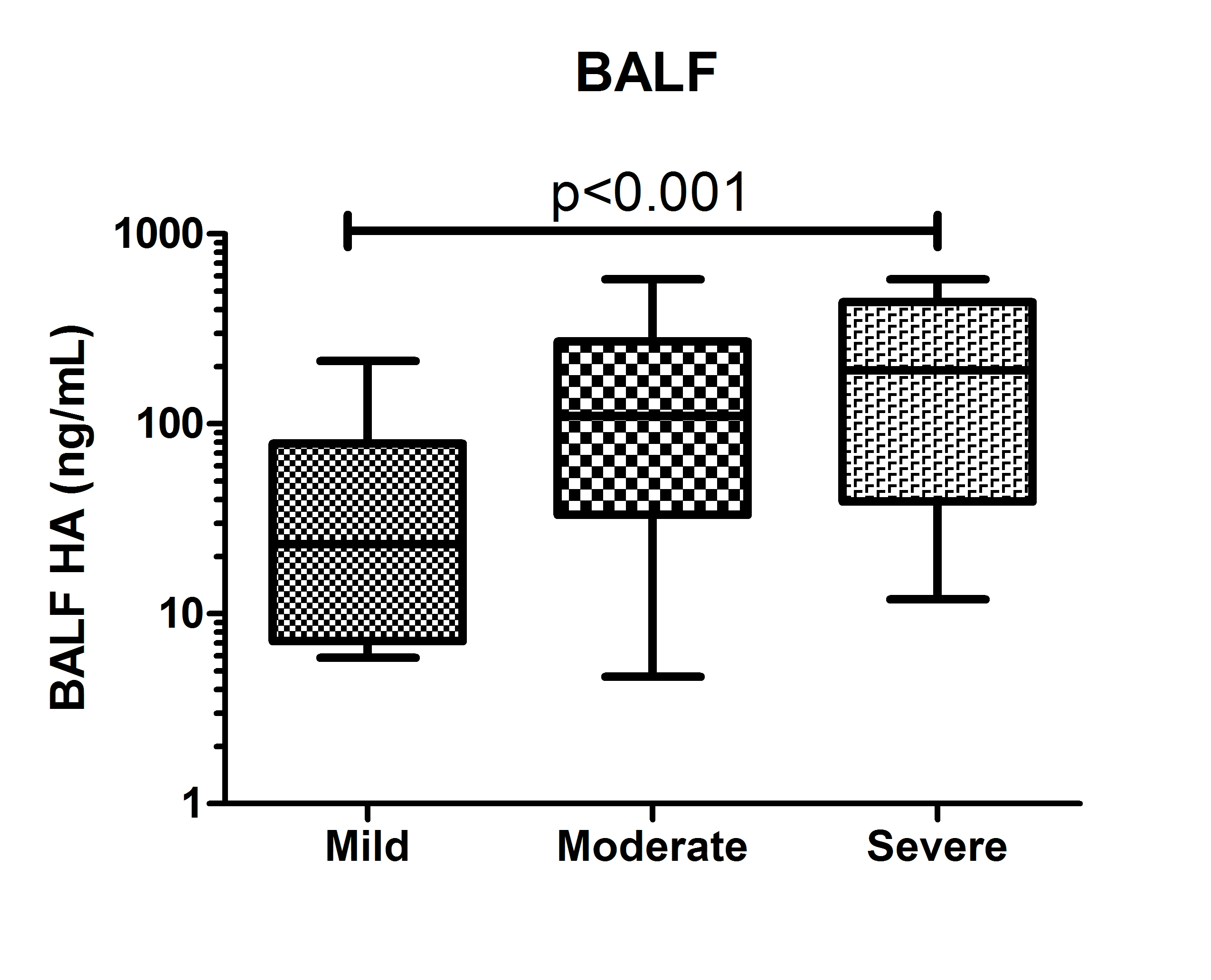


**A)**


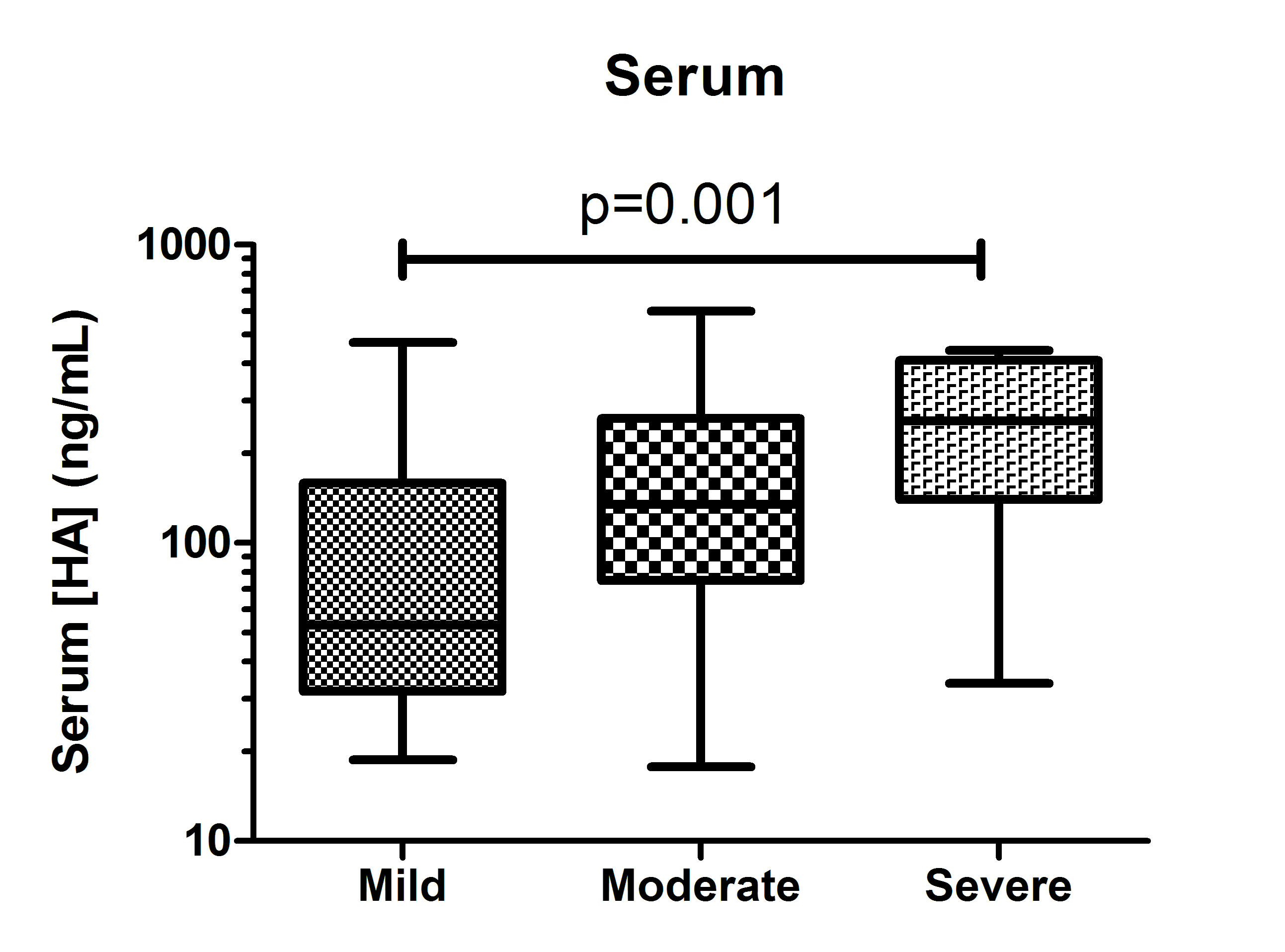


**B)**

Additional File 4. BALF HA (A) and serum HA (B) are associated with severity of ARDS by the Berlin Criteria. BALF HA and serum HA were measured on day 0 in patients with ARDS. The p value represents an association between HA and severity of ARDS using multiple linear regression adjusting for age, gender, race, treatment group (placebo vs fish oil), and ARDS risk factor (direct vs indirect). Mild ARDS is defined by the Berlin Criteria of PaO2/FiO2 of <=300 and >200, Moderate ARDS PaO2/FiO2 of <=200 and >100, and Severe ARDS of PaO2/FiO2 <=100.
