# Supplementary material for: Hyaluronic acid is associated with organ dysfunction in acute respiratory distress syndrome
Source: Crit Care. 2017 Dec 14;21:304. doi: 10.1186/s13054-017-1895-7 (PMC5729515; doi:10.1186/s13054-017-1895-7)
Supplement: Supplementary file 5 — Both circulating and alveolar hyaluronic acid (HA) levels are associated with hypoxemia (C, D) and the set positive end-expiratory pressure (PEEP) (E, F), but not the involved quadrants on a chest radiograph (CXR) (A, B) or respiratory system compliance (G, H), components of the lung injury score (LIS). This figure provides the reader with graphical representation and corresponding analysis of the reported data in Table 3 of the main text. (DOCX 259 kb) [file 13054_2017_1895_MOESM5_ESM.docx]

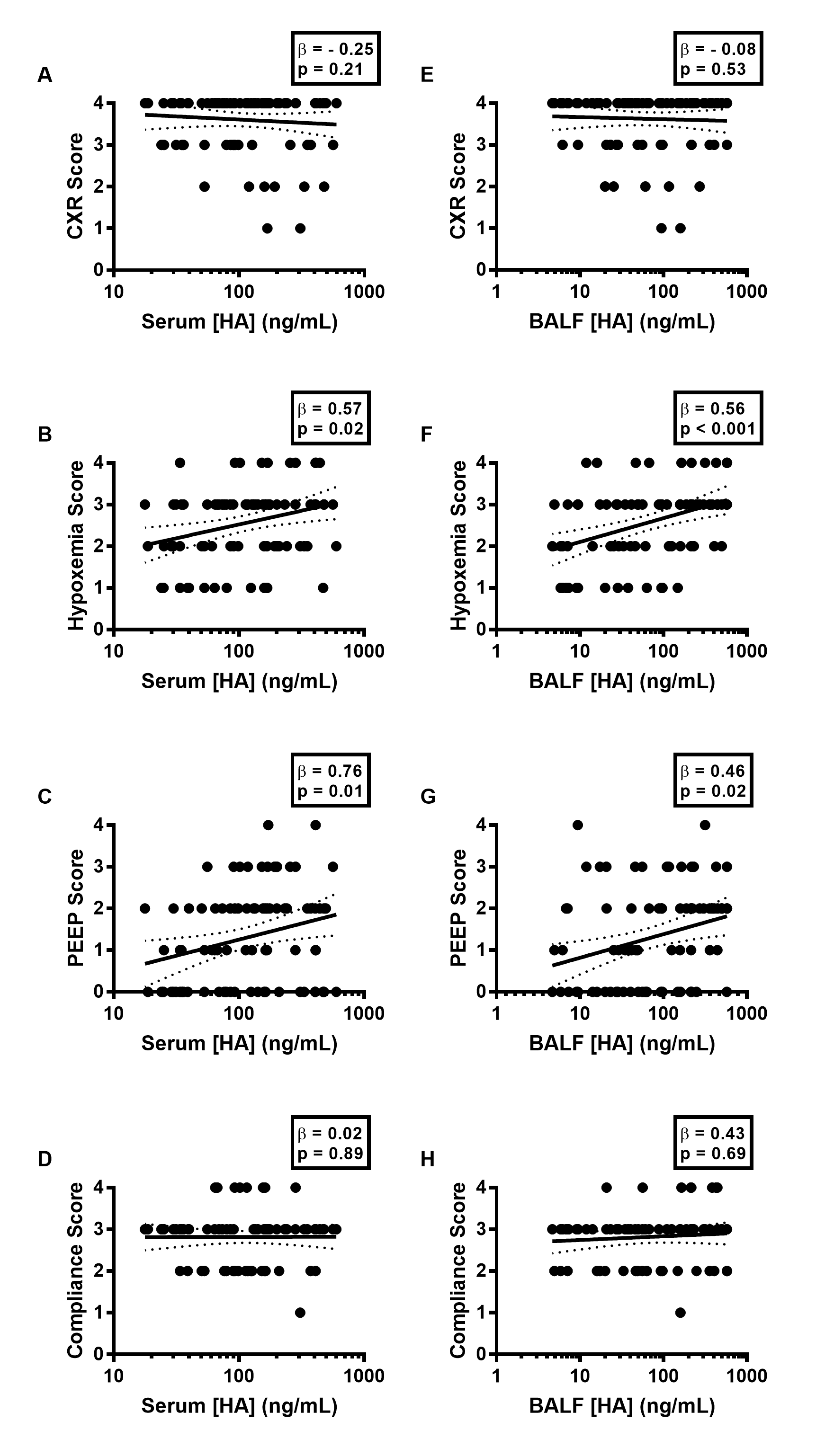


**Additional File 5.** **Both Circulating and alveolar hyaluronic acid (HA) levels are associated with the hypoxemia (C, D) and set positive end-expiratory pressure (PEEP; E, F), but not the involved quadrants on a chest radiograph (CXR; A, B) or respiratory system compliance (G, H), components of the lung injury score (LIS).** [HA] = concentration of hyaluronic acid. β values represent a change in units of each LIS component score per 10-fold increase in [HA]. Solid lines represent regression lines determined via linear regression analyses while hashed lines represent the 95% confidence interval of the regression line. See Additional File 1 for information regarding calculation of composite LIS.
