# Supplementary material for: Hyaluronic acid is associated with organ dysfunction in acute respiratory distress syndrome
Source: Crit Care. 2017 Dec 14;21:304. doi: 10.1186/s13054-017-1895-7 (PMC5729515; doi:10.1186/s13054-017-1895-7)
Supplement: Supplementary file 6 — Early (Day 0) hyaluronic acid levels are not associated with mortality. This table provides the reader with analysis of the association between circulating and alveolar hyaluronic acid levels and 28-day mortality. (DOCX 16 kb) [file 13054_2017_1895_MOESM6_ESM.docx]

**Additional File 6. Early (Day 0) Hyaluronic Acid Levels are Not Associated with Mortality.**

| **Outcome** | **Source** | **OR (95% CI)*^a^*** | **p** | **OR (95% CI)*^b^*** | **p** |
| --- | --- | --- | --- | --- | --- |
| 28-Day Mortality | Serum | 3.44 (0.70 – 16.88) | 0.13 | 3.15 (0.53 – 18.70) | 0.21 |
|  | BALF*^c^* | 0.59 (0.23 – 1.56) | 0.29 | 0.57 (0.19 – 1.73) | 0.32 |

*^a^* Logistic regression between log_10_ transformed day 0 HA concentration and 28-day mortality. OR = odds ratio.

*^b^* Logistic regression adjusted for age, sex, race, treatment group, and ARDS etiology.

*^c^* Two patients did not have day 0 BALF samples (*n* = 84).
