# Supplementary material for: Hyaluronic acid is associated with organ dysfunction in acute respiratory distress syndrome
Source: Crit Care. 2017 Dec 14;21:304. doi: 10.1186/s13054-017-1895-7 (PMC5729515; doi:10.1186/s13054-017-1895-7)
Supplement: Supplementary file 7 — Both circulating (A) and alveolar (B) HA levels are associated with the respiratory component of the sequential organ failure assessment (SOFA) score, while circulatory, but not alveolar, HA levels are also associated with coagulation (C, D), and liver (E, F) components. Circulatory, but not alveolar, HA levels are also associated with renal components (K, L). Neither is associated with the cardiovascular (G, H) or neurologic (I, J) component. This figure provides the reader with graphical representation and corresponding analysis of the reported data in Table 4 of the text. (DOCX 486 kb) [file 13054_2017_1895_MOESM7_ESM.docx]

**
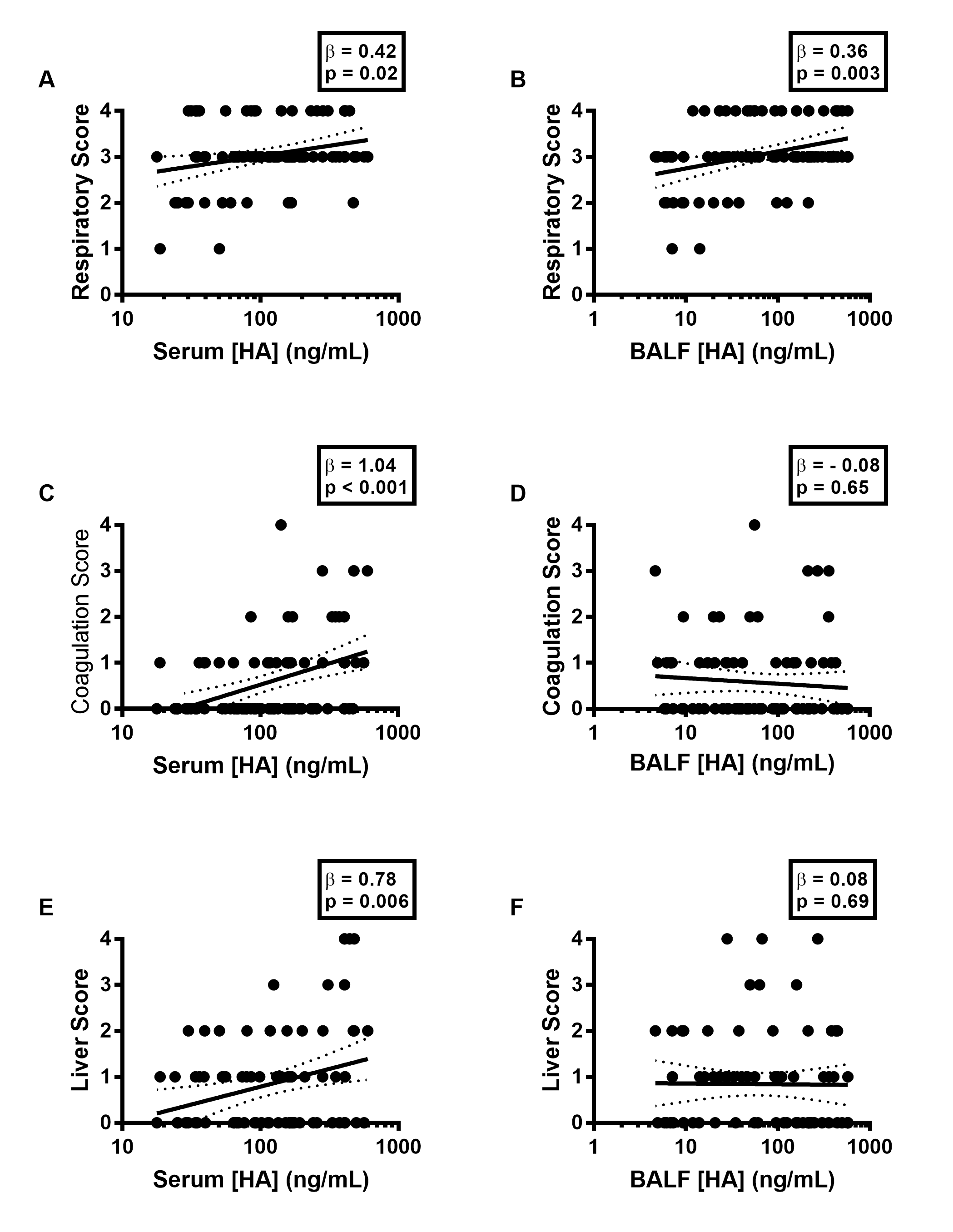
**

**Additional File 7.** **Both circulating (A) and alveolar (B) HA levels are associated with the respiratory component of the sequential organ failure assessment (SOFA) score, while circulatory, but not alveolar, HA levels are also associated with the coagulation (C, D), liver (E, F) components.**

*
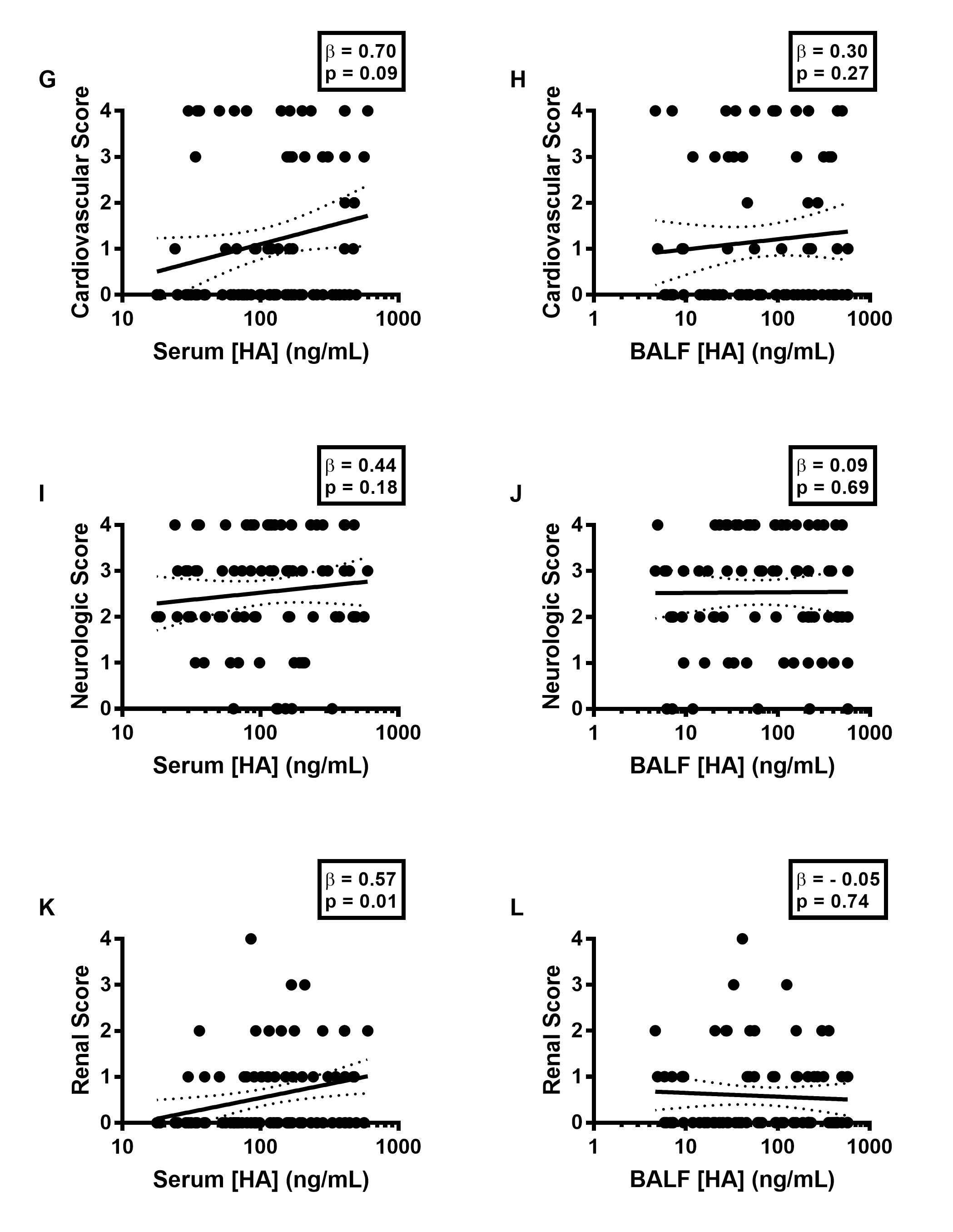
*

**Additional File 7 cont’d.** **Circulatory, but not alveolar, HA levels are also associated with renal components (K, L). Neither is associated with the cardiovascular (G, H) or neurologic (I, J) component.** [HA] = concentration of hyaluronic acid. β values represent a change in units of each SOFA component score per 10-fold increase in [HA]. Solid lines represent regression lines determined via linear regression analyses while hashed lines represent the 95% confidence interval of the regression line. See Additional File 2 for information regarding calculation of composite SOFA score.
